# The genome sequence of Reeves’ muntjac, *Muntiacus reevesi *(Ogilby, 1839)

**DOI:** 10.12688/wellcomeopenres.22608.1

**Published:** 2024-07-10

**Authors:** Nick Ewart, Denise C. Wawman, University of Oxford and Wytham Woods Genome Acquisition Lab, Darwin Tree of Life Barcoding collective, Wellcome Sanger Institute Tree of Life Management, Samples and Laboratory team, Wellcome Sanger Institute Scientific Operations: Sequencing Operations, Wellcome Sanger Institute Tree of Life Core Informatics team, Tree of Life Core Informatics collective, Darwin Tree of Life Consortium

**Affiliations:** 1 Wytham Woods, University of Oxford, Oxford, England, UK; 2 Edward Grey Institute, Department of Biology, University of Oxford, Oxford, England, UK

**Keywords:** Muntiacus reevesi, Reeves' muntjac, genome sequence, chromosomal, Artiodactyla

## Abstract

We present a genome assembly from an individual female *Muntiacus reevesi* (the Reeves’ muntjac; Chordata; Mammalia; Artiodactyla; Cervidae). The genome sequence is 2,656.2 megabases in span. Most of the assembly is scaffolded into 23 chromosomal pseudomolecules, including the X sex chromosome. The mitochondrial genome has also been assembled and is 16.35 kilobases in length.

## Species taxonomy

Eukaryota; Opisthokonta; Metazoa; Eumetazoa; Bilateria; Deuterostomia; Chordata; Craniata; Vertebrata; Gnathostomata; Teleostomi; Euteleostomi; Sarcopterygii; Dipnotetrapodomorpha; Tetrapoda; Amniota; Mammalia; Theria; Eutheria; Boreoeutheria; Laurasiatheria; Artiodactyla; Ruminantia; Pecora; Cervidae; Muntiacinae; *Muntiacus*; *Muntiacus reevesi reevesi* (Ogilby, 1839) (NCBI:txid9886).

## Background

The Reeve’s Muntjac or Chinese Muntjac *Muntiacus reevesi reevesi* is a small deer in the family Cervidae. They are 0.44 to 0.52 metres tall at the shoulder. Full grown males weigh 10 to 18 kg and females 9 to 16 kg ( British Deer Society, 2024). Males have a distinctive pattern of facial stripes and small anthers that do not branch and usually curve inwards ( British Deer Society, 2024).

The Reeve’s Muntjac is native to the forests, grassland and shrubland of south-east China but has been introduced into Europe: the first pair brought from China to London Zoo in 1838 being described as the type specimens ( Chapman, 2021). Multiple other introductions followed, and Reeve’s Muntjac were released in various counties including Bedfordshire, Oxfordshire, Northamptonshire, Kent, Devon, and on the Norfolk/Suffolk border. By 1994 this subspecies was present in over half of England and Wales, and has continued to spread ( Chapman, 2021). It is also present in Northern Ireland and the Republic of Ireland ( Chapman, 2021). This species’ ecology means that the population can increase rapidly: males are fertile by the time they are nine months old and females, which can give birth at any time of year, are fertile at five to six months of age ( Chapman, 2021).

Although numbers are declining in its native range, Reeve’s Muntjac has been ranked at number 9 in a list of the “100 worst alien species in Europe” based on their impact on the native ecology ( Nentwig *et al.*, 2018). By overgrazing young coppice and the understorey of more mature woodland, Reeve’s Muntjac may, with other species of deer, be contributing to habitat changes resulting in the decline of some bird species ( Fuller *et al.*, 2005; Gill & Fuller, 2007). They may be partly responsible for the possible increase in the range of the tick *Ixodes ricinus* ( Gandy *et al.*, 2023). Reeve’s Muntjac cause damage to timber crops, allotments and gardens, and increasing numbers of road traffic accidents ( British Deer Society, 2024).

We present a chromosomally complete genome sequence for a female *Muntiacus reevesi reevesi*, based on one specimen collected in Wytham Woods as part of the Darwin Tree of Life Project.

## Genome sequence report

**Figure 1.**
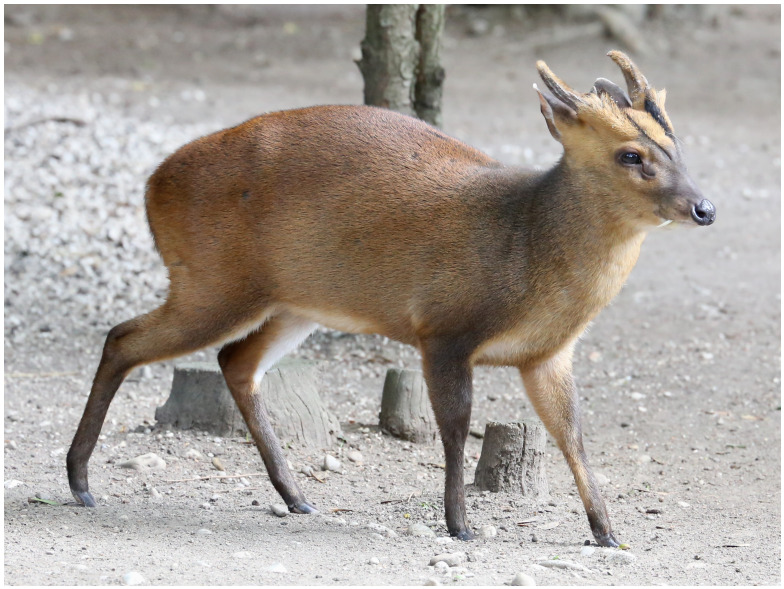
Photograph of *Muntiacus reevesi* by Rufus46 (not the specimen used for genome sequencing).

The genome was sequenced from adult female *Muntiacus reevesi* ( Figure 1) collected from Wytham Woods, Oxfordshire, UK (51.77, –1.34). A total of 42-fold coverage in Pacific Biosciences single-molecule HiFi long reads was generated. Primary assembly contigs were scaffolded with chromosome conformation Hi-C data. Manual assembly curation corrected 20 missing joins or mis-joins and removed one haplotypic duplication, reducing the scaffold number by 3.60%, and increasing the scaffold N50 by 0.77%.

**Figure 2.**
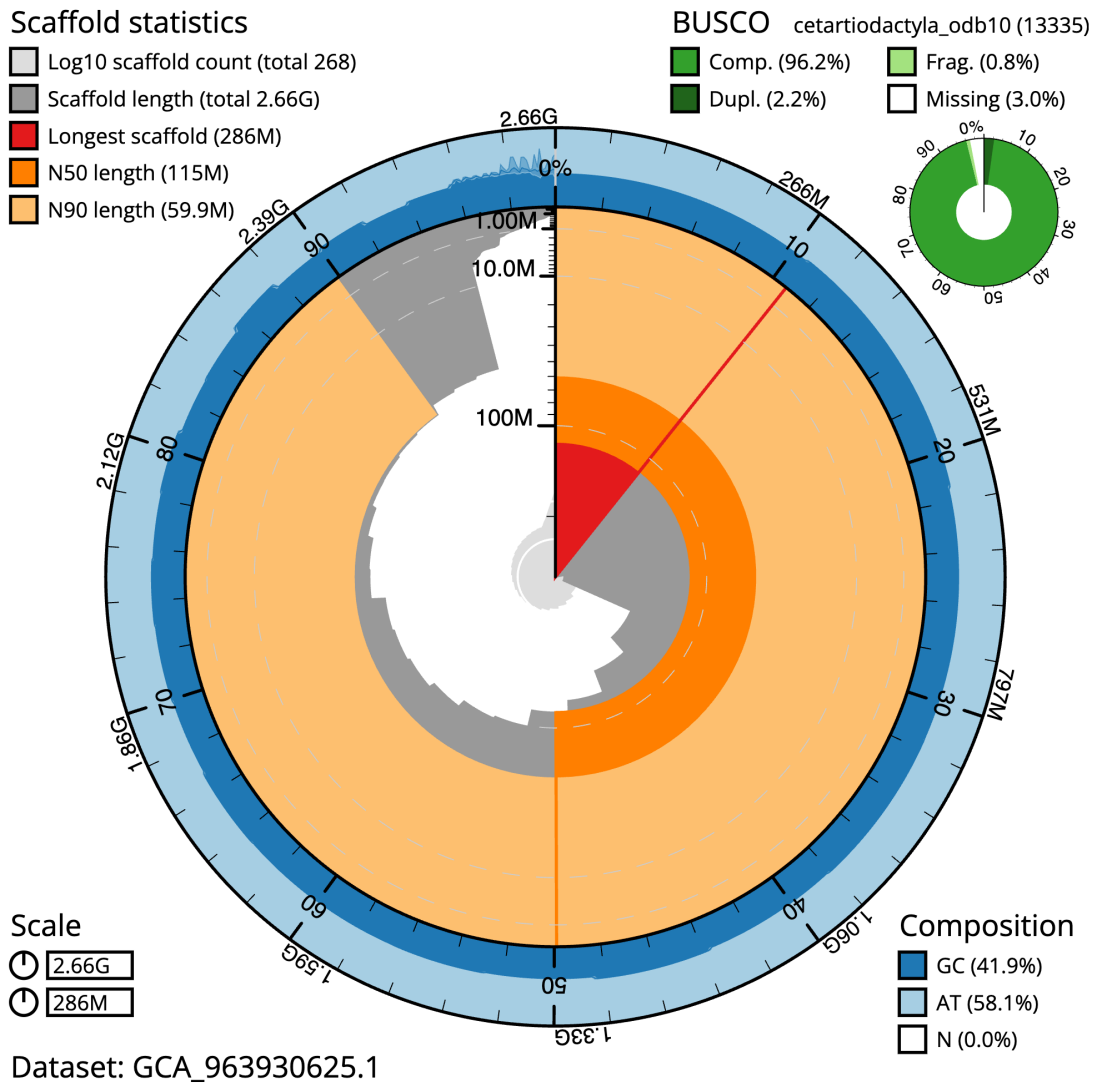
Genome assembly of *Muntiacus reevesi*, mMunRee1.1: metrics. The BlobToolKit snail plot shows N50 metrics and BUSCO gene completeness. The main plot is divided into 1,000 size-ordered bins around the circumference with each bin representing 0.1% of the 2,656,184,892 bp assembly. The distribution of scaffold lengths is shown in dark grey with the plot radius scaled to the longest scaffold present in the assembly (285,828,854 bp, shown in red). Orange and pale-orange arcs show the N50 and N90 scaffold lengths (114,544,513 and 59,907,558 bp), respectively. The pale grey spiral shows the cumulative scaffold count on a log scale with white scale lines showing successive orders of magnitude. The blue and pale-blue area around the outside of the plot shows the distribution of GC, AT and N percentages in the same bins as the inner plot. A summary of complete, fragmented, duplicated and missing BUSCO genes in the cetartiodactyla_odb10 set is shown in the top right. An interactive version of this figure is available at https://blobtoolkit.genomehubs.org/view/Muntiacus_reevesi/dataset/GCA_963930625.1/snail.

**Figure 3.**
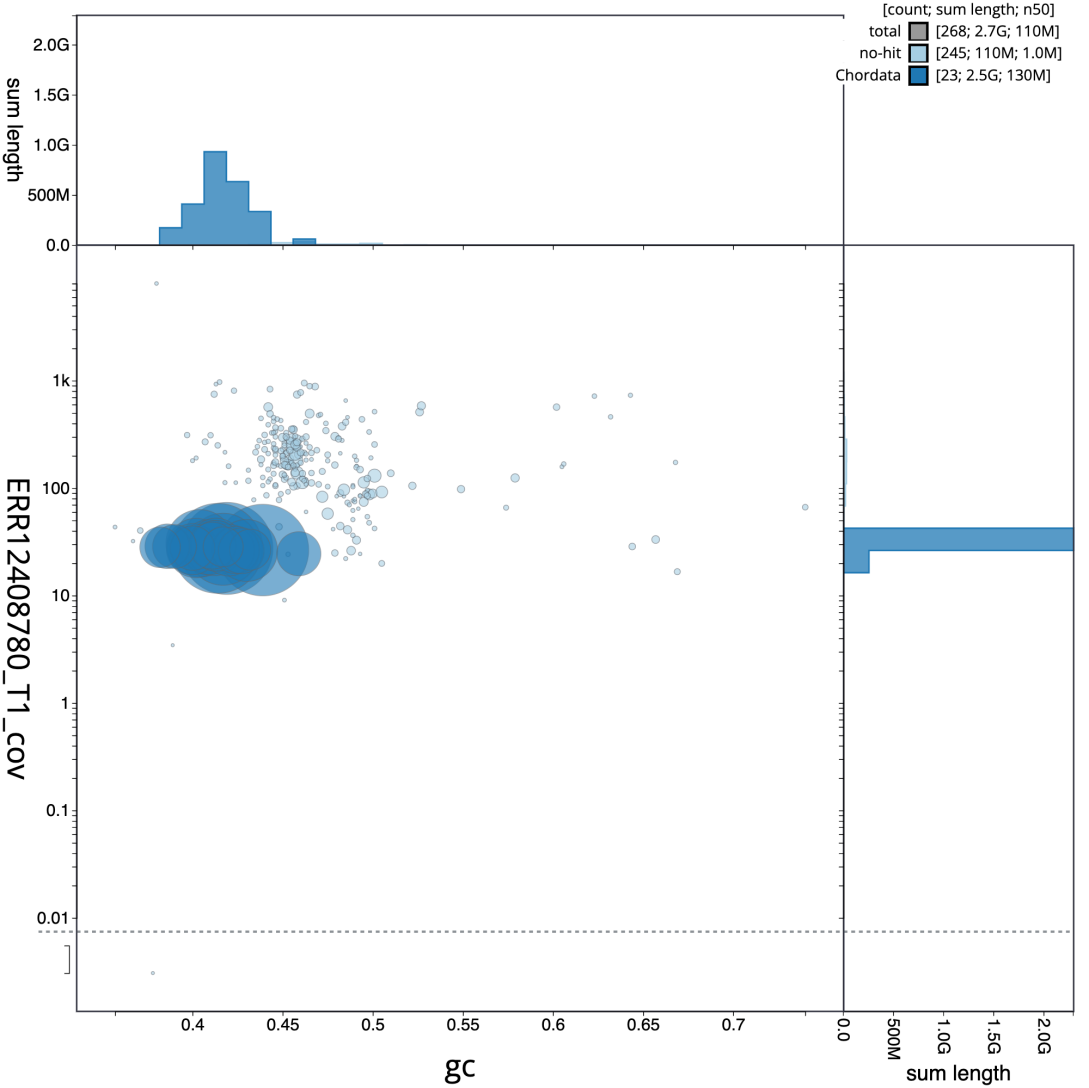
Genome assembly of *Muntiacus reevesi*, mMunRee1.1: BlobToolKit GC-coverage plot. Sequences are coloured by phylum. Circles are sized in proportion to sequence length. Histograms show the distribution of sequence length sum along each axis. An interactive version of this figure is available at https://blobtoolkit.genomehubs.org/view/Muntiacus_reevesi/dataset/GCA_963930625.1/blob.

**Figure 4.**
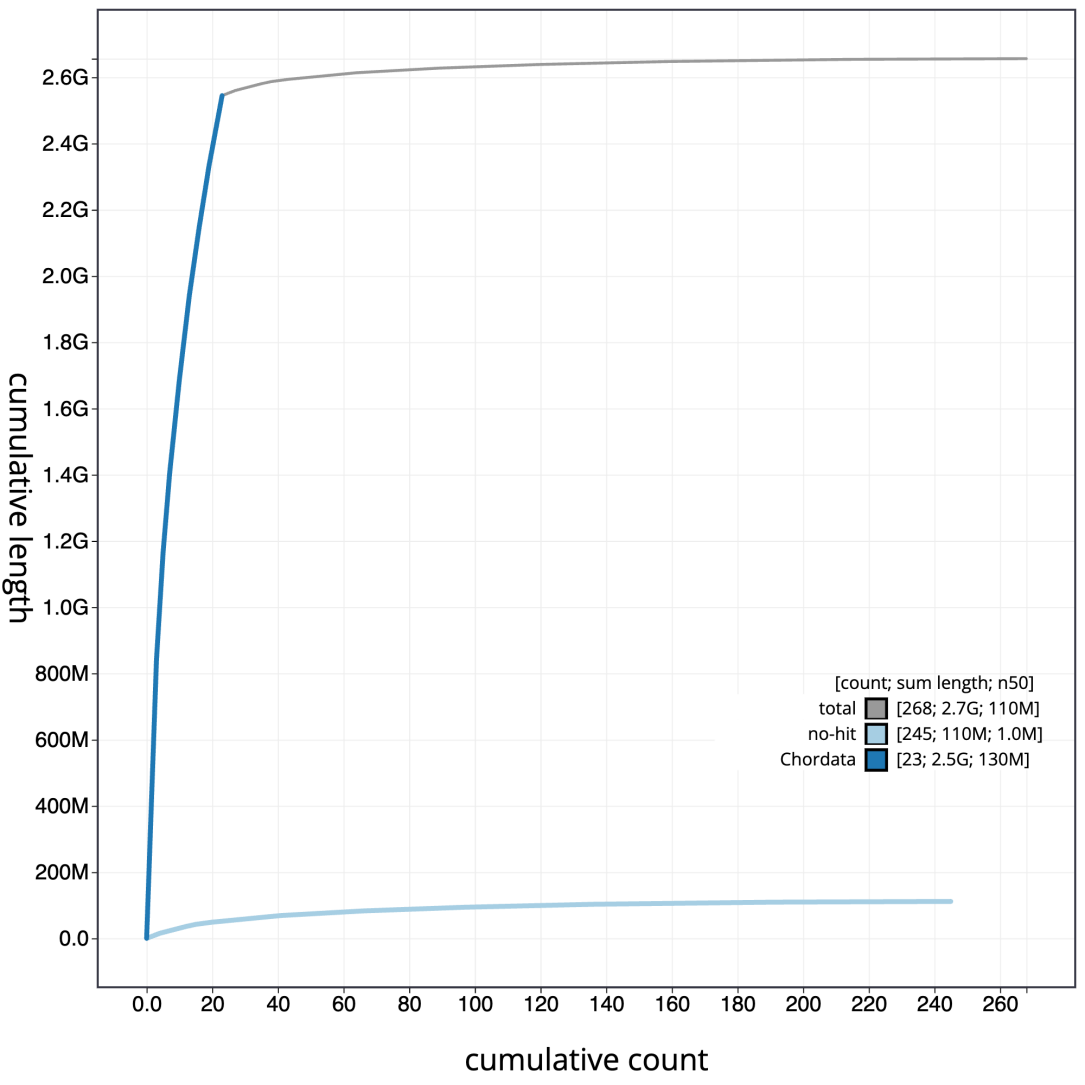
Genome assembly of *Muntiacus reevesi* mMunRee1.1: BlobToolKit cumulative sequence plot. The grey line shows cumulative length for all sequences. Coloured lines show cumulative lengths of sequences assigned to each phylum using the buscogenes taxrule. An interactive version of this figure is available at https://blobtoolkit.genomehubs.org/view/Muntiacus_reevesi/dataset/GCA_963930625.1/cumulative.

**Figure 5.**
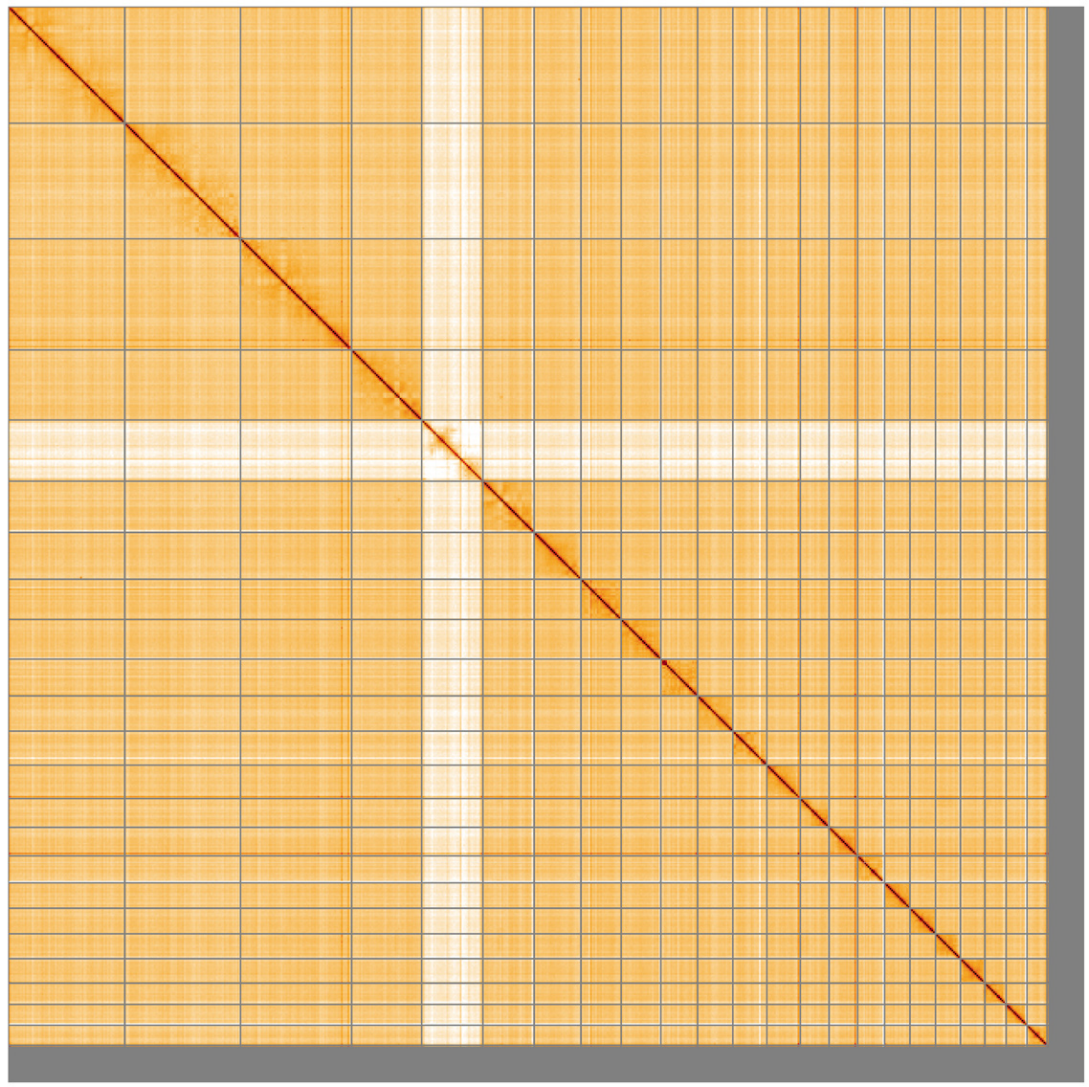
Genome assembly of *Muntiacus reevesi* mMunRee1.1: Hi-C contact map of the mMunRee1.1 assembly, visualised using HiGlass. Chromosomes are shown in order of size from left to right and top to bottom. An interactive version of this figure may be viewed at https://genome-note-higlass.tol.sanger.ac.uk/l/?d=C-2GvvVfStOlDyKBbe-IFQ.

The final assembly has a total length of 2,656.2 Mb in 267 sequence scaffolds with a scaffold N50 of 114.5 Mb ( Table 1). The snail plot in Figure 2 provides a summary of the assembly statistics, while the distribution of assembly scaffolds on GC proportion and coverage is shown in Figure 3. The cumulative assembly plot in Figure 4 shows curves for subsets of scaffolds assigned to different phyla. Most (96.02%) of the assembly sequence was assigned to 23 chromosomal-level scaffolds, representing 22 autosomes and the X sex chromosome. Chromosome-scale scaffolds confirmed by the Hi-C data are named in order of size ( Figure 5; Table 2). The X chromosome was identified based on synteny with *Muntiacus reevesi* (GCA_020226045.1). While not fully phased, the assembly deposited is of one haplotype. Contigs corresponding to the second haplotype have also been deposited. The mitochondrial genome was also assembled and can be found as a contig within the multifasta file of the genome submission.

**Table 1.** Genome data for *Muntiacus reevesi*, mMunRee1.1.

Project accession data		
Assembly identifier	mMunRee1.1	
Species	*Muntiacus reevesi*	
Specimen	mMunRee1	
NCBI taxonomy ID	9886	
BioProject	PRJEB71430	
BioSample ID	Genome and RNA sequencing: SAMEA110690974 Hi-C scaffolding: SAMEA110690979	
Isolate information	mMunRee1, female: muscle (DNA and RNA sequencing) mMunRee2, male: muscle (Hi-C sequencing)	
Assembly metrics *		*Benchmark*
Consensus quality (QV)	68.2	*≥ 50*
*k*-mer completeness	100.0%	*≥ 95%*
BUSCO **	C:96.1%[S:93.9%,D:2.2%], F:0.9%,M:3.0%,n:13,335	*C ≥ 95%*
Percentage of assembly mapped to chromosomes	96.02%	*≥ 95%*
Sex chromosomes	XX	*localised homologous pairs*
Organelles	Mitochondrial genome: 16.35 kb	*complete single alleles*
Raw data accessions		
PacificBiosciences Revio	ERR12408780, ERR12408781, ERR12408782, ERR12408783	
Hi-C Illumina	ERR12512727	
PolyA RNA-Seq Illumina	ERR12512728	
Genome assembly		
Assembly accession	GCA_963930625.1	
*Accession of alternate haplotype*	GCA_963930665.1	
Span (Mb)	2,656.2	
Number of contigs	950	
Contig N50 length (Mb)	5.8	
Number of scaffolds	267	
Scaffold N50 length (Mb)	114.5	
Longest scaffold (Mb)	285.83	

* Assembly metric benchmarks are adapted from column VGP-2020 of “Table 1: Proposed standards and metrics for defining genome assembly quality” from Rhie *et al.* (2021). ** BUSCO scores based on the cetartiodactyla_odb10 BUSCO set using version 5.4.3. C = complete [S = single copy, D = duplicated], F = fragmented, M = missing, n = number of orthologues in comparison. A full set of BUSCO scores is available at https://blobtoolkit.genomehubs.org/view/Muntiacus_reevesi/dataset/GCA_963930625.1/busco.

**Table 2.** Chromosomal pseudomolecules in the genome assembly of *Muntiacus reevesi*, mMunRee1.

INSDC accession	Chromosome	Length (Mb)	GC%
OZ005624.1	1	285.83	42.0
OZ005625.1	2	283.02	44.0
OZ005626.1	3	272.12	41.5
OZ005627.1	4	171.98	41.5
OZ005629.1	5	125.49	43.0
OZ005630.1	6	114.54	40.0
OZ005631.1	7	98.9	41.0
OZ005632.1	8	96.94	41.0
OZ005633.1	9	90.45	41.5
OZ005634.1	10	85.65	42.0
OZ005635.1	11	83.45	40.0
OZ005636.1	12	81.91	41.5
OZ005637.1	13	70.73	42.5
OZ005638.1	14	69.96	41.0
OZ005639.1	15	65.76	42.5
OZ005640.1	16	62.46	38.5
OZ005641.1	17	62.23	40.0
OZ005642.1	18	61.04	46.0
OZ005643.1	19	59.91	39.0
OZ005644.1	20	52.4	43.0
OZ005645.1	21	50.89	38.0
OZ005646.1	22	49.36	41.5
OZ005628.1	X	149.6	40.5
OZ005647.1	MT	0.02	38.0

The estimated Quality Value (QV) of the final assembly is 68.2 with *k*-mer completeness of 100.0%, and the assembly has a BUSCO v5.4.3 completeness of 96.1% (single = 93.9%, duplicated = 2.2%), using the cetartiodactyla_odb10 reference set ( *n* = 13,335).

Metadata for specimens, barcode results, spectra estimates, sequencing runs, contaminants and pre-curation assembly statistics are given at https://tolqc.cog.sanger.ac.uk/darwin/mammals/Muntiacus_reevesi/

## Methods

### Sample acquisition and nucleic acid extraction

The samples used in this study were taken from two *Muntiacus reevesi* individuals culled in Wytham Woods, Oxfordshire (biological vice-county Berkshire), UK (latitude 51.77, longitude –1.34) on 2022-03-24. The animals were collected and identified by Nick Ewart (University of Oxford), and tissue samples were stored on dry ice. Samples from the female (specimen ID Ox002445, ToLID mMunRee1) were used for genome and RNA sequencing, while samples from the male (specimen ID Ox002446, ToLID mMunRee2) were used for Hi-C scaffolding.

The workflow for high molecular weight (HMW) DNA extraction at the Wellcome Sanger Institute (WSI) Tree of Life Core Laboratory includes a sequence of core procedures: sample preparation; sample homogenisation, DNA extraction, fragmentation, and clean-up. In sample preparation, the mMunRee1 sample was weighed and dissected on dry ice ( Jay *et al.*, 2023). For sample homogenisation, muscle tissue was cryogenically disrupted using the Covaris cryoPREP ^®^ Automated Dry Pulverizer ( Narváez-Gómez *et al.*, 2023). HMW DNA was extracted using the Manual MagAttract v1 protocol ( Strickland *et al.*, 2023b). DNA was sheared into an average fragment size of 12–20 kb in a Megaruptor 3 system with speed setting 30 ( Todorovic *et al.*, 2023). Sheared DNA was purified by solid-phase reversible immobilisation ( Strickland *et al.*, 2023a): in brief, the method employs a 1.8X ratio of AMPure PB beads to sample to eliminate shorter fragments and concentrate the DNA. The concentration of the sheared and purified DNA was assessed using a Nanodrop spectrophotometer and Qubit Fluorometer and Qubit dsDNA High Sensitivity Assay kit. Fragment size distribution was evaluated by running the sample on the FemtoPulse system.

RNA was extracted from muscle tissue of mMunRee1 in the Tree of Life Laboratory at the WSI using the RNA Extraction: Automated MagMax™ *mir*Vana protocol ( do Amaral *et al.*, 2023). The RNA concentration was assessed using a Nanodrop spectrophotometer and a Qubit Fluorometer using the Qubit RNA Broad-Range Assay kit. Analysis of the integrity of the RNA was done using the Agilent RNA 6000 Pico Kit and Eukaryotic Total RNA assay.

Protocols developed by the WSI Tree of Life laboratory are publicly available on protocols.io ( Denton *et al.*, 2023).

### Sequencing

Pacific Biosciences HiFi circular consensus DNA sequencing libraries were constructed according to the manufacturers’ instructions. Poly(A) RNA-Seq libraries were constructed using the NEB Ultra II RNA Library Prep kit. DNA and RNA sequencing was performed by the Scientific Operations core at the WSI on Pacific Biosciences Revio (HiFi) and Illumina NovaSeq 6000 (RNA-Seq) instruments. Hi-C data were also generated from muscle tissue of mMunRee2 using the Arima2 kit and sequenced on the Illumina NovaSeq 6000 instrument.

### Genome assembly and curation

Assembly was carried out with Hifiasm ( Cheng *et al.*, 2021) and haplotypic duplication was identified and removed with purge_dups ( Guan *et al.*, 2020). The assembly was then scaffolded with Hi-C data ( Rao *et al.*, 2014) using YaHS ( Zhou *et al.*, 2023). The assembly was checked for contamination and corrected using the TreeVal pipeline ( Pointon *et al.*, 2023). Manual curation was performed using JBrowse2 ( Diesh *et al.*, 2023), HiGlass ( Kerpedjiev *et al.*, 2018) and PretextView ( Harry, 2022). The mitochondrial genome was assembled using MitoHiFi ( Uliano-Silva *et al.*, 2023), which runs MitoFinder ( Allio *et al.*, 2020) or MITOS ( Bernt *et al.*, 2013) and uses these annotations to select the final mitochondrial contig and to ensure the general quality of the sequence.

### Evaluation of final assembly

The final assembly was post-processed and evaluated with the three Nextflow ( Di Tommaso *et al.*, 2017) DSL2 pipelines “sanger-tol/readmapping” ( Surana *et al.*, 2023a), “sanger-tol/genomenote” ( Surana *et al.*, 2023b), and “sanger-tol/blobtoolkit” ( Muffato *et al.*, 2024). The pipeline sanger-tol/readmapping aligns the Hi-C reads with bwa-mem2 ( Vasimuddin *et al.*, 2019) and combines the alignment files with SAMtools ( Danecek *et al.*, 2021). The sanger-tol/genomenote pipeline transforms the Hi-C alignments into a contact map with BEDTools ( Quinlan & Hall, 2010) and the Cooler tool suite ( Abdennur & Mirny, 2020), which is then visualised with HiGlass ( Kerpedjiev *et al.*, 2018). It also provides statistics about the assembly with the NCBI datasets ( Sayers *et al.*, 2024) report, computes *k*-mer completeness and QV consensus quality values with FastK and MerquryFK, and a completeness assessment with BUSCO ( Manni *et al.*, 2021).

The sanger-tol/blobtoolkit pipeline is a Nextflow port of the previous Snakemake Blobtoolkit pipeline ( Challis *et al.*, 2020). It aligns the PacBio reads with SAMtools and minimap2 ( Li, 2018) and generates coverage tracks for regions of fixed size. In parallel, it queries the GoaT database ( Challis *et al.*, 2023) to identify all matching BUSCO lineages to run BUSCO ( Manni *et al.*, 2021). For the three domain-level BUSCO lineage, the pipeline aligns the BUSCO genes to the Uniprot Reference Proteomes database ( Bateman *et al.*, 2023) with DIAMOND ( Buchfink *et al.*, 2021) blastp. The genome is also split into chunks according to the density of the BUSCO genes from the closest taxonomically lineage, and each chunk is aligned to the Uniprot Reference Proteomes database with DIAMOND blastx. Genome sequences that have no hit are then chunked with seqtk and aligned to the NT database with blastn ( Altschul *et al.*, 1990). All those outputs are combined with the blobtools suite into a blobdir for visualisation.

All three pipelines were developed using the nf-core tooling ( Ewels *et al.*, 2020), use MultiQC ( Ewels *et al.*, 2016), and make extensive use of the Conda package manager, the Bioconda initiative ( Grüning *et al.*, 2018), the Biocontainers infrastructure ( da Veiga Leprevost *et al.*, 2017), and the Docker ( Merkel, 2014) and Singularity ( Kurtzer *et al.*, 2017) containerisation solutions.

Table 3 contains a list of relevant software tool versions and sources.

**Table 3.** Software tools: versions and sources.

Software tool	Version	Source
BEDTools	2.30.0	https://github.com/arq5x/bedtools2
Blast	2.14.0	ftp://ftp.ncbi.nlm.nih.gov/blast/executables/blast+/
BlobToolKit	4.3.7	https://github.com/blobtoolkit/blobtoolkit
BUSCO	5.4.3 and 5.5.0	https://gitlab.com/ezlab/busco
bwa-mem2	2.2.1	https://github.com/bwa-mem2/bwa-mem2
Cooler	0.8.11	https://github.com/open2c/cooler
DIAMOND	2.1.8	https://github.com/bbuchfink/diamond
fasta_windows	0.2.4	https://github.com/tolkit/fasta_windows
FastK	427104ea91c78c3b8b8b49f1a7d6bbeaa869ba1c	https://github.com/thegenemyers/FASTK
GoaT CLI	0.2.5	https://github.com/genomehubs/goat-cli
Hifiasm	0.19.5-r587	https://github.com/chhylp123/hifiasm
HiGlass	1.11.6	https://github.com/higlass/higlass
HiGlass	44086069ee7d4d3f6f3f0012569789ec138f42b84a a44357826c0b6753eb28de	https://github.com/higlass/higlass
MerquryFK	d00d98157618f4e8d1a9190026b19b471055b22e	https://github.com/thegenemyers/MERQURY.FK
MitoHiFi	3	https://github.com/marcelauliano/MitoHiFi
MultiQC	1.14, 1.17, and 1.18	https://github.com/MultiQC/MultiQC
NCBI Datasets	15.12.0	https://github.com/ncbi/datasets
Nextflow	23.04.0-5857	https://github.com/nextflow-io/nextflow
PretextView	0.2	https://github.com/wtsi-hpag/PretextView
purge_dups	1.2.5	https://github.com/dfguan/purge_dups
samtools	1.16.1, 1.17, and 1.18	https://github.com/samtools/samtools
sanger-tol/genomenote	1.1.1	https://github.com/sanger-tol/genomenote
sanger-tol/readmapping	1.2.1	https://github.com/sanger-tol/readmapping
Seqtk	1.3	https://github.com/lh3/seqtk
Singularity	3.9.0	https://github.com/sylabs/singularity
TreeVal	1.0.0	https://github.com/sanger-tol/treeval
YaHS	1.2a.2	https://github.com/c-zhou/yahs

### Wellcome Sanger Institute – Legal and Governance

The materials that have contributed to this genome note have been supplied by a Darwin Tree of Life Partner. The submission of materials by a Darwin Tree of Life Partner is subject to the **‘Darwin Tree of Life Project Sampling Code of Practice’**, which can be found in full on the Darwin Tree of Life website here. By agreeing with and signing up to the Sampling Code of Practice, the Darwin Tree of Life Partner agrees they will meet the legal and ethical requirements and standards set out within this document in respect of all samples acquired for, and supplied to, the Darwin Tree of Life Project. 

Further, the Wellcome Sanger Institute employs a process whereby due diligence is carried out proportionate to the nature of the materials themselves, and the circumstances under which they have been/are to be collected and provided for use. The purpose of this is to address and mitigate any potential legal and/or ethical implications of receipt and use of the materials as part of the research project, and to ensure that in doing so we align with best practice wherever possible. The overarching areas of consideration are:

• Ethical review of provenance and sourcing of the material

• Legality of collection, transfer and use (national and international) 

Each transfer of samples is further undertaken according to a Research Collaboration Agreement or Material Transfer Agreement entered into by the Darwin Tree of Life Partner, Genome Research Limited (operating as the Wellcome Sanger Institute), and in some circumstances other Darwin Tree of Life collaborators.

## Data Availability

European Nucleotide Archive: *Muntiacus reevesi* (Reeves' muntjac). Accession number PRJEB71430; https://identifiers.org/ena.embl/PRJEB71430 ( Wellcome Sanger Institute, 2024). The genome sequence is released openly for reuse. The *Muntiacus reevesi* genome sequencing initiative is part of the Darwin Tree of Life (DToL) project. All raw sequence data and the assembly have been deposited in INSDC databases. The genome will be annotated using available RNA-Seq data and presented through the Ensembl pipeline at the European Bioinformatics Institute. Raw data and assembly accession identifiers are reported in Table 1.

## Author information

Members of the University of Oxford and Wytham Woods Genome Acquisition Lab are listed here: https://doi.org/10.5281/zenodo.7125292.

Members of the Darwin Tree of Life Barcoding collective are listed here: https://doi.org/10.5281/zenodo.4893703.

Members of the Wellcome Sanger Institute Tree of Life Management, Samples and Laboratory team are listed here: https://doi.org/10.5281/zenodo.10066175.

Members of Wellcome Sanger Institute Scientific Operations: Sequencing Operations are listed here: https://doi.org/10.5281/zenodo.10043364.

Members of the Wellcome Sanger Institute Tree of Life Core Informatics team are listed here: https://doi.org/10.5281/zenodo.10066637.

Members of the Tree of Life Core Informatics collective are listed here: https://doi.org/10.5281/zenodo.5013541.

Members of the Darwin Tree of Life Consortium are listed here: https://doi.org/10.5281/zenodo.4783558.
