# Supplementary material for: The genome sequence of Reeves’ muntjac, Muntiacus reevesi (Ogilby, 1839)
Source: Wellcome Open Res. 2024 Jul 10;9:368. [Version 1] doi: 10.12688/wellcomeopenres.22608.1 (PMC11427868; doi:10.12688/wellcomeopenres.22608.1)
Supplement: Peer review history

# Reviewer report: Reviewer response for version 1

**DOI:** 10.21956/wellcomeopenres.24907.r98515

**Published:** 2024-10-04

**Authors:** Kun Wang

**Affiliations:** 1 School of Ecology and Environment, Northwestern Polytechnical University, Xi’an, China

The manuscript appears well-written and detailed description of the sequencing, assembly, curation, and evaluation processes for the Muntiacus reevesi genome. The text demonstrates a high level of scientific rigor and transparency in describing the methodologies used. 

 Small issues:

 1. Background:"small anthers"? Is it small antlers?

 2. There is both Reeves' muntjac and Reeve's muntjac. It should be unified.

 3. Methods: "and Qubit Fluorometer and Qubit dsDNA High Sensitivity Assay kit." can be replaced by "and a Qubit Fluorometer with the Qubit dsDNA High Sensitivity Assay kit."

Are sufficient details of methods and materials provided to allow replication by others?

Yes

Is the rationale for creating the dataset(s) clearly described?

Yes

Are the datasets clearly presented in a useable and accessible format?

Yes

Are the protocols appropriate and is the work technically sound?

Yes

Reviewer Expertise:

Evolutionary genomics

I confirm that I have read this submission and believe that I have an appropriate level of expertise to confirm that it is of an acceptable scientific standard.

## References

[1] : Merqury: reference-free quality, completeness, and phasing assessment for genome assemblies. *Genome Biol* .2020;21(1) : 10.1186/s13059-020-02134-9 245 10.1186/s13059-020-02134-9 32928274 PMC7488777

# Reviewer report: Reviewer response for version 1

**DOI:** 10.21956/wellcomeopenres.24907.r98518

**Published:** 2024-10-02

**Authors:** Josephine Pemberton

**Affiliations:** 1 The University of Edinburgh, Edinburgh, Scotland, UK

This is a neat and succinct report of the work to assemble the genome of the Chinese or Reeve’s muntjac, an introduced deer species to the UK. To the extent that I can judge the work has been thorough and extremely carefully QC’d prior to indexing.

 All my specific comments relate to the Background section

 Para 1 end:

 In mature males there can be a small branch at the base of the antlers.

 Suggest also mention that both sexes have tusks which are used by males in fights.

 Para 2 end:

 Worth saying that individual mature females breed every 7 months and unlike in other deer species, males are fertile irrespective of the antler cycle (ref Chapman 2021).

 Para 4 end:

 It would be worth mentioning that the related Indian muntjac ( *Muntiacus muntjak*) is known for its extraordinarily low chromosome number (females 2N=6 and males 2N=7) and there is much interest in the chromosome fusions that have taken place en route to the Indian muntjac - see ref here: https://www.nature.com/articles/s42003-020-1096-9 .  It seems to me this publication should be referred to regardless, given it describes an earlier assembly for *reevesi *(currently it is not cited) *. *As should any other previous genome sequences for this species.

 Figure 1:

 Legend could point out that the tusk is visible.

Are sufficient details of methods and materials provided to allow replication by others?

Yes

Is the rationale for creating the dataset(s) clearly described?

Yes

Are the datasets clearly presented in a useable and accessible format?

Yes

Are the protocols appropriate and is the work technically sound?

Yes

Reviewer Expertise:

Ecology and Evolution

I confirm that I have read this submission and believe that I have an appropriate level of expertise to confirm that it is of an acceptable scientific standard.

# Reviewer report: Reviewer response for version 1

**DOI:** 10.21956/wellcomeopenres.24907.r98516

**Published:** 2024-09-26

**Authors:** Frasier Timothy

**Affiliations:** 1 Professor of Biology and Forensic Science, Saint Mary's University, Halifax, Canada

**GENERAL**

 This manuscript describes the development of a chromosomal-length genome sequence of Reeves' Muntjac, using both Pac-Bio and Illumina methods; as well as RNA sequencing data. The work seems very well done, and is presented quite clearly. I am impressed with the open and reproducible nature of the work; particularly that protocols are available on protocols.io, and sample metadata available on Tree of Life QC. The table of software and versions used is also a very clear and helpful way to present this information. Overall, it is a very clear paper, and I imagine these sequences will be useful for a wide range of researchers.

**SPECIFIC**

 1. I answered "No" to the question "Is the rationale for creating the dataset(s) clearly described" because no such rationale was presented. The authors give a nice review of the history and status of Reeve's Muntjac, which was quite interesting, but they do not describe their rationale for sequencing this genome and providing it as a resource. It would therefore be helpful for the authors to add a sentence or two describing their motivation for doing so.

 2. It would be helpful to have a little more context for the culling of the individuals used. The authors are clear about this work meeting ethical guidelines and things like that; but for those of us not familiar, a sentence or two about the context of this culling would helpful. For example, is there a regular cull of some individuals for population control, and these individuals were part of that process? Or, perhaps the collector just had a license for hunting, and the individuals were killed particularly for this work? Regardless, such context would help readers sleep easier regarding the situation within which these individuals were culled and these samples collected.

Are sufficient details of methods and materials provided to allow replication by others?

Yes

Is the rationale for creating the dataset(s) clearly described?

No

Are the datasets clearly presented in a useable and accessible format?

Yes

Are the protocols appropriate and is the work technically sound?

Yes

Reviewer Expertise:

Conservation genomics

I confirm that I have read this submission and believe that I have an appropriate level of expertise to confirm that it is of an acceptable scientific standard.

# Reviewer report: Reviewer response for version 1

**DOI:** 10.21956/wellcomeopenres.24907.r91839

**Published:** 2024-08-08

**Authors:** Jayan D.M Senevirathna

**Affiliations:** 1 Uva Wellassa University, Badulla, Uva Province, Sri Lanka

Excellent work and can be accepted with minor modifications.

 Minor suggestions are as follows.

- In the abstract; mention only the X chromosome
- Keywords; scientific names should be Italic.
- Background; Please mention if there are distinguish characters in female, *M. reevesi* and importance of genome sequencing of this species.
- Genome sequence report; Location coordinates no need to mention here as it is included in the methodology. Reduce repetitions.
- Methods; Mention the maturity level of the animal and voucher number of the sample if you stored at the museum. 
- Include and specify how did you use RNA data in the genome analysis.

Are sufficient details of methods and materials provided to allow replication by others?

Yes

Is the rationale for creating the dataset(s) clearly described?

Yes

Are the datasets clearly presented in a useable and accessible format?

Yes

Are the protocols appropriate and is the work technically sound?

Yes

Reviewer Expertise:

Genomics

I confirm that I have read this submission and believe that I have an appropriate level of expertise to confirm that it is of an acceptable scientific standard.
